# AXL Promotes Metformin-Induced Apoptosis Through Mediation of Autophagy by Activating ROS-AMPK-ULK1 Signaling in Human Esophageal Adenocarcinoma

**DOI:** 10.3389/fonc.2022.903874

**Published:** 2022-07-22

**Authors:** Jun Hong, Selma Maacha, Nataliya Pidkovka, Andreia Bates, Safia N. Salaria, Mary K. Washington, Abbes Belkhiri

**Affiliations:** ^1^ Department of Surgery, Vanderbilt University Medical Center, Nashville, TN, United States; ^2^ Division of Translational Medicine, Sidra Medicine, Doha, Qatar; ^3^ Department of Health Science, South College, Nashville, TN, United States; ^4^ Department of Pathology, Microbiology and Immunology, Vanderbilt University Medical Center, Nashville, TN, United States; ^5^ Vanderbilt-Ingram Cancer Center, Vanderbilt University Medical Center, Nashville, TN, United States

**Keywords:** AMPK, autophagic flux, ATG7, Barrett’s esophagus, Beclin1, glucose starvation, proliferation, reactive oxygen species

## Abstract

AXL receptor tyrosine kinase promotes an invasive phenotype and chemotherapy resistance in esophageal adenocarcinoma (EAC). AXL has been implicated in the regulation of autophagy, but the underlying molecular mechanism remains poorly understood. Herein, we investigate the mechanistic role of AXL in autophagy as well as metformin-induced effects on the growth and survival of EAC. We demonstrate that AXL mediates autophagic flux through activation of AMPK-ULK1 signaling in a reactive oxygen species (ROS)-dependent mechanism by glucose starvation. AXL positively regulates basal cellular ROS levels without significantly affecting mitochondrial ROS production in EAC cells. Pharmacological inhibition of cellular ROS using Trolox abrogates glucose starvation-induced AMPK signaling and autophagy. We demonstrate that AXL expression is required for metformin-induced apoptosis in EAC cells *in vitro*. The apoptosis induction by metformin is markedly attenuated by inhibition of autophagy through genetic silencing of Beclin1 or ATG7 autophagy mediators, thereby confirming the requirement of intact autophagy for enhancing metformin-induced apoptosis in EAC cells. Our data indicate that metformin-induced autophagy displays a pro-apoptotic function in EAC cells. We show that the metformin-induced suppression of tumor growth *in vivo* is highly dependent on AXL expression in a tumor xenograft mouse model of EAC. We demonstrate that AXL promotes metformin-induced apoptosis through activation of autophagy in EAC. AXL may be a valuable biomarker to identify tumors that are sensitive to metformin. Therefore, AXL expression could inform the selection of patients for future clinical trials to evaluate the therapeutic efficacy of metformin in EAC.

## Introduction

Esophageal adenocarcinoma (EAC) is a highly aggressive malignancy with a poor prognosis due to its late-stage diagnosis and limited treatment options (1). Intriguingly, the incidence of EAC is markedly increasing in Western countries where it has become the most prevalent histological type of esophageal cancer (2). Gastroesophageal reflux disease and Barrett’s esophagus (BE) are considered the most important risk factors for the development of EAC due to their induced long-term chronic damage of the esophageal mucosa (3, 4). Overexpression of AXL receptor tyrosine kinase has been associated with Barrett’s tumorigenesis and poor prognosis in EAC (5). AXL promotes resistance to chemotherapy (6–8) and enhances cell invasion through the regulation of extracellular acidification and lysosome trafficking in EAC cells (9).

Macro-autophagy (hereafter autophagy), which is one type of three autophagic processes including micro-autophagy and chaperone-mediated autophagy, is an intracellular self-degradative process that maintains cellular homeostasis and metabolism by recycling cellular material through lysosomal degradation. Autophagy is induced in response to nutrients or growth factors limitation, hypoxia, or oncogenic and oxidative stresses (10). Accumulation of reactive oxygen species (ROS) is triggered upon nutrient deprivation, inducing autophagy or cell death (11). The production of ROS in tumor cells effectively induces autophagy through activation of the energy sensor AMP-activated protein kinase (AMPK) (12, 13). Activation of AXL by its ligand GAS6 has been shown to induce autophagy in macrophages through MAPK14 activity in an acute liver injury mouse model (14). Another study reported that AXL kinase inhibition by small molecule inhibitor bemcentinib inhibited the autophagic flux and induced immunogenic cell death in drug-resistant non-small cell lung cancer *in vitro* (15). However, a prior study revealed that cell treatment with bemcentinib suppressed autophagy in an AXL-independent mechanism in glioblastoma cells (16). Although these studies clearly demonstrate that AXL positively regulates autophagy, the underlying molecular mechanism remains poorly understood in cancer cells. Autophagy is tightly controlled by coordinated phosphorylation of UNC51-like Kinase 1 (ULK1) and its binding to AMPK. Under glucose starvation, ULK1 is activated by AMPK through phosphorylation of Ser317, Ser467, Ser555, Ser777, and Thr574 residues resulting in the induction of autophagy (17). Conversely, under nutrients sufficiency, autophagy is inhibited by a high mammalian target of Rapamycin (mTOR) activity that prevents the interaction between ULK1 and AMPK through phosphorylation of Ser757 in ULK1 (18). Autophagy is considered as a cell survival mechanism but also displays a tumor-suppressive function (19). In a recent study, inhibition of autophagy by the lysosome inhibitor chloroquine or genetic silencing of ATG7 enhanced the cytotoxic effect of cisplatin in ovarian cancer cells, implying the pro-survival function of autophagy (20). Accumulating evidence shows that treatments inducing ROS production trigger autophagic cell death in cancer cells (21, 22). Controversially, autophagy exhibits both tumor-promoting and tumor-suppressive functions in cancer cells. The regulation of autophagy is cancer type-dependent as well as context-dependent (23). Findings suggest that in premalignant lesions, inducing autophagy may prevent cancer development, whereas, once malignant lesions are established, increasing autophagy may promote tumor cells survival and growth (24). Specifically, in the case of EAC, low levels of autophagy are described in dysplastic BE and EAC tumors, whereas higher levels are observed in squamous epithelium and non-dysplastic BE (25). Given the dismal clinical outcome of EAC, manipulation of autophagy could improve response to therapies.

Metformin, the most common biguanide used in the treatment of type 2 diabetes, regulates cancer cell proliferation, autophagy, and cell death in different types of cancers (26–29). Specifically, metformin disrupts cancer cell energy metabolism through activation of AMPK, inducing autophagy and apoptosis. A previous study showed that metformin selectively inhibited cell growth of esophageal squamous cell carcinoma (ESCC), and pharmacological or genetic inhibition of autophagy sensitized ESCC cells to metformin-induced apoptosis (30). Additionally, metformin has been shown to reduce mRNA and protein expression of AXL and TYRO3 receptor tyrosine kinases to overcome chemoresistance in human ovarian cancer cells (31). Notably, retrospective epidemiological studies showed that diabetes patients have decreased cancer incidence with metformin (32, 33).

Here, we report that AXL mediates autophagy as well as metformin-induced apoptosis through activation of ROS-AMPK-ULK1 signaling in EAC cells. Our novel findings indicate that AXL-dependent autophagy is required for the induction of apoptosis by metformin in EAC cells. These data strongly suggest that AXL expression may be a promising predictive biomarker of response to metformin treatment in EAC.

## Results

### AXL Mediates Autophagic Flux in EAC Cells

**Figure 1:**
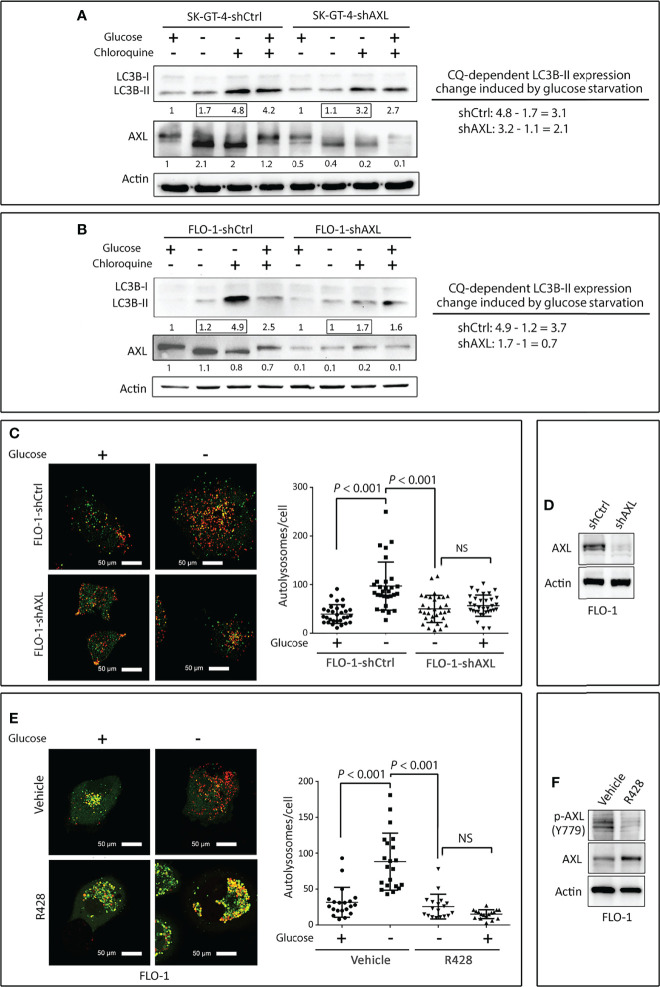
Downregulation of AXL expression impairs autophagic flux in EAC cells. **(A**, **B)** SK-GT-4 and FLO-1 cells stably expressing control shRNA or AXL shRNA were cultured in complete medium with 4.5 g/L glucose or without glucose in the presence or absence of the lysosomal inhibitor chloroquine (10 µM) for 4 h. Western blot analysis of LC3B and AXL proteins in whole cell lysates from SK-GT-4-shCtrl and SK-GT-4-shAXL **(A)** and FLO-1-shCtrl and FLO-1-shAXL **(B)** cells. Gel loading was normalized for equal β-actin. Autophagic flux was assessed based on LC3B-II expression change induced by chloroquine following glucose starvation. The LC3B-II expression change was calculated by subtracting quantified LC3B-II value of cells treated with chloroquine from that of cells without chloroquine following glucose starvation. **(C)** FLO-1-shCtrl and FLO-1-shAXL cells stably expressing mRFP-GFP tandem fluorescent-tagged LC3 reporter (tfLC3) were cultured in the presence or absence of glucose for 4 h to induce autophagy. Representative confocal images (60x) of live cells depicting autophagosomes (green fluorescence) and autolysosomes (red fluorescence). Quantification of total number of autolysosomes per cell in FLO-1-shCtrl *versus* FLO-1-shAXL cells. Data are represented as median ± SD. **(D)** Western blot analysis of AXL in whole cell lysates from FLO-1-shCtrl and FLO-1-shAXL cells. **(E)** Representative confocal images (60x) of live FLO-1-tfLC3 stable cells showing autophagosomes (green puncta) and autolysosomes (red puncta) following treatment with vehicle or AXL inhibitor R428 (500 nM) in the absence or presence of glucose for 4 h. Quantification of total number of autolysosomes per cell in vehicle-treated FLO-1 *versus* R428-treated FLO-1 cells. Data are represented as median ± SD. **(F)** Western blot analysis of p-AXL (Y779) and AXL proteins in whole cell lysates from FLO-1 cells treated with vehicle (DMSO) or R428 (500 nM). Data are representative of three independent experiments and differences were analyzed by one-way ANOVA followed by the Newman-Keuls *post hoc* test. NS, statistically not significant.

Solid tumors including EAC are regularly subjected to shortages of oxygen and nutrients, and in response to these harsh conditions, tumors activate the survival and adaptive mechanisms that comprise autophagy (34, 35). However, excessive autophagy could induce cell death (36, 37). We investigated if AXL regulates autophagy in EAC cells. SK-GT-4 and FLO-1 cells stably expressing control shRNA or AXL shRNA were cultured in 4.5 g/L glucose or without glucose in the presence or absence of the lysosome inhibitor chloroquine (CQ, 10 µM) for 4 h. We found that the knockdown of AXL expression reduced the accumulation of LC3B-II proteins in in SK-GT-4 cells (**Figure 1A**) and FLO-1 cells (**Figure 1B**) in response to glucose starvation. The autophagic flux was determined by the change in LC3B-II protein level induced by glucose starvation in the presence or absence of CQ. Based on the interpretation of LC3B immunoblotting by Mizushima (38), the rise of LC3B-II protein level reflects the increase in autophagic flux.

To confirm that AXL mediates autophagic flux, we investigated the role of AXL in autolysosomes formation using mRFP-GFP tandem fluorescent-tagged LC3 plasmid reporter (tfLC3). Formation of autolysosomes, which involves autophagosomes fusion with lysosomes, is a critical step that precedes autophagic flux. FLO-1-shCtrl and FLO-1-shAXL cells stably expressing tfLC3 reporter were cultured in the presence or absence of glucose for 4 h, and autolysosomes formation was evaluated by confocal microscopy. We found that glucose starvation significantly induced autolysosomes formation, as indicated by the quantity of red puncta per cell, in FLO-1-shCtrl cells relative to FLO-1-shAXL cells (*P* < 0.001, **Figure 1C**). Knockdown of AXL expression in FLO-1 cells was verified by Western blot analysis (**Figure 1D**). Additionally, inhibition of AXL with R428 (0.5 µM) in FLO-1-tfLC3 cells significantly suppressed autolysosomes formation as compared to vehicle-treated cells in response to glucose starvation (*P* < 0.001, **Figure 1E**). AXL inhibition with R428 in FLO-1 cells was confirmed by immunoblotting (**Figure 1F**).

We next investigated if AXL expression is required for inducing autophagic flux by inhibition of mTOR pathway with rapamycin as an alternative approach to glucose starvation. FLO-1-shCtrl cells and FLO-1-shAXL cells stably expressing tfLC3 were cultured in the presence of rapamycin (100 nM) or vehicle for 4 h, and autolysosomes formation was assessed by confocal fluorescence microscopy. Indeed, we found that knocking down AXL expression significantly suppressed autolysosomes formation, as indicated by red puncta, in response to rapamycin (*P* < 0.01, **Figure S1**). Collectively, these results demonstrate that AXL expression is required for mediating autophagic flux in response to glucose starvation or inhibition of the mTOR pathway in EAC cells.

### AXL Mediates Activation of AMPK and Autophagy in EAC Cells

**Figure 2:**
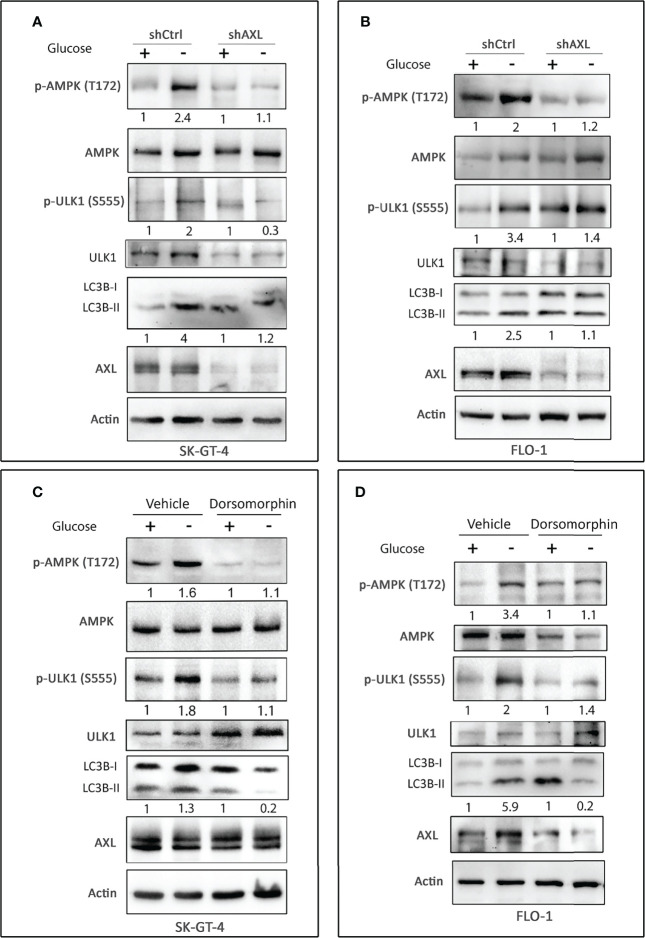
AXL expression and AMPK activation are required for inducing autophagy in EAC cells. Western blot analysis of p-AMPK (T172), AMPK, p-ULK1 (S555), ULK1, LC3B, and AXL proteins in whole cell lysates from SK-GT-4-shCtrl and SK-GT-4-shAXL cells **(A)** and FLO-1-shCtrl and FLO-1-shAXL cells **(B)** cultured in complete medium with and without glucose for 4 h to induce autophagy. Western blot analysis of p-AMPK (T172), AMPK, p-ULK1 (S555), ULK1, LC3B, and AXL in cell lysates from SK-GT-4 **(C)** and FLO-1 **(D)** cells following treatment with vehicle or AMPK inhibitor dorsomorphin (5 µM) in the presence or absence of glucose for 4 h. Gel loading was normalized for equal β-actin.

AMPK, a sensor of cellular energy, when activated by glucose starvation, induces autophagy through regulation of downstream signaling (39, 40). We investigated if AXL expression is required for phosphorylation and activation of AMPK by glucose starvation in EAC cells. Indeed, we found that AXL knockdown markedly decreased the induction of p-AMPK (T172), p-ULK1 (S555), and LC3B-II protein levels in response to glucose starvation for 4 h in SK-GT-4 cells (**Figure 2A**) and FLO-1 cells (**Figure 2B**) relative to control cells. Interestingly, the basal expression levels of p-ULK1 (S555) and LC3B-II proteins were higher in AXL-knockdown cells than in control cells under normal culture conditions (**Figures 2A, B**). These effects could be induced by a compensatory mechanism in response to knockdown of AXL expression. We next investigated whether AMPK activation is required for the phosphorylation of the serine/threonine protein kinase ULK1, which plays a central role in autophagy. We cultured cells with or without glucose and in the presence or absence of the AMPK specific inhibitor, dorsomorphin (5 µM), for 4 h. The data indicated that AMPK inhibition markedly reduced the induction of p-ULK1 (S555) and LC3B-II protein levels in response to glucose starvation in SK-GT-4 cells (**Figure 2C**) and FLO-1 cells (**Figure 2D**). Together, the results demonstrated that AXL mediates glucose starvation-induced autophagy through regulation of AMPK-ULK1 signaling in EAC cells.

### Cellular ROS Mediate Autophagy Through Regulation of AMPK-ULK1 Signaling in EAC Cells

**Figure 3:**
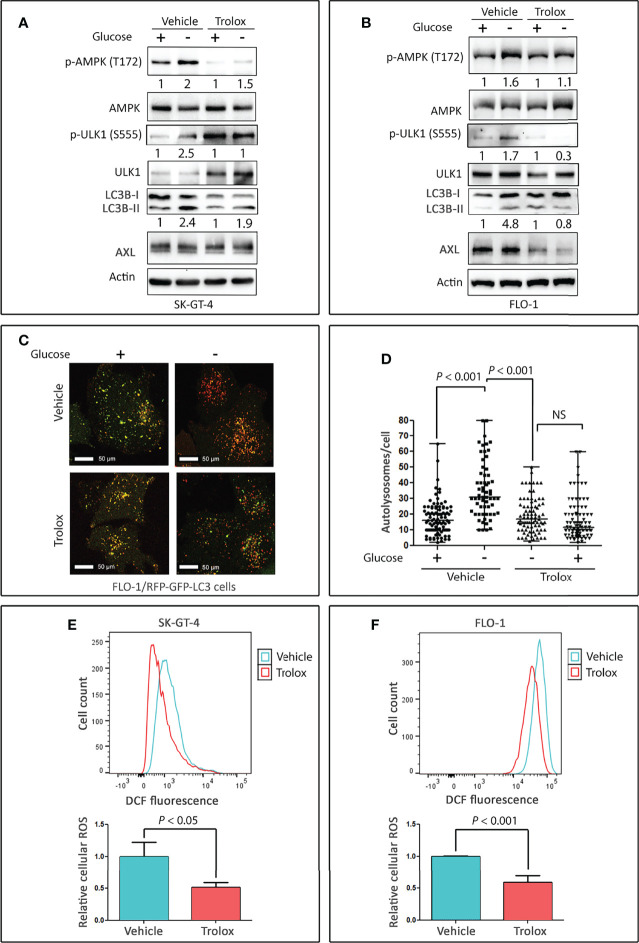
Cellular ROS mediate activation of AMPK and autophagy in EAC cells. Western blot analysis of p-AMPK (T172), AMPK, p-ULK1 (S555), ULK1, LC3B, and AXL proteins in whole cell lysates from SK-GT-4 cells **(A)** and FLO-1 cells **(B)** following treatment with vehicle or 100 µM Trolox, a vitamin E analog and ROS scavenger, in the presence or absence of glucose for 4 h. FLO-1 cells stably expressing mRFP-GFP tandem fluorescent-tagged LC3 reporter (tfLC3) were cultured in the presence or absence of glucose and treated with vehicle or 100 µM Trolox for 4 h to induce autophagy. **(C)** Representative confocal images (60x) of live cells depicting autophagosomes (green puncta) and autolysosomes (red puncta). Quantification of total number of autolysosomes per cell. **(D)** Data are shown as median ± SD. Cellular ROS levels in SK-GT-4 cells **(E)** and FLO-1 cells **(F)** were evaluated by DCFDA/H2DCFDA – Cellular Reactive Oxygen Species Detection Assay Kit and flow cytometry following treatment with vehicle or 100 µM Trolox for 4 h. Cells were gated using forward scatter and side scatter to remove debris and dead cells, and 10^4^ live cell events were recorded. To quantify changes in DCFDA signal, mean fluorescent intensity after gating was used. The relative fluorescence was calculated by normalizing the mean fluorescent intensity of treated cells to that of control cells. Data are representative of three independent experiments and statistical significance was evaluated by one-way ANOVA followed by the Newman-Keuls *post hoc* test.

As ROS induce autophagy through several mechanisms involving Atg4, catalase, and mitochondrial electron transport chain in cancer cells (reviewed in (41)), we investigated if ROS mediate induction of autophagy in EAC cells. SK-GT-4 and FLO-1 cells were cultured for 4 h with or without glucose and in the presence of vehicle (DMSO) or 100 µM Trolox (a vitamin E analog and scavenger that reduces cellular ROS levels). The results showed that treatment with Trolox markedly decreased the induction of p-AMPK (T172), p-ULK1 (S555), and LC3B-II protein levels in SK-GT-4 cells (**Figure 3A**) and FLO-1 cells (**Figure 3B**) in response to glucose starvation relative to control cells. Interestingly, treatment with Trolox under non-starving conditions substantially decreased p-AMPK (T172) and LC3B-II basal levels and increased ULK1 and p-ULK1 (S555) proteins in SK-GT-4 cells (**Figure 3B**). These effects may be induced by a decrease in basal ROS levels. We next investigated if inhibition of cellular ROS could suppress autolysosomes formation, a step required for autophagic flux. FLO-1 cells stably expressing tfLC3 were cultured in the presence or absence of glucose with vehicle or Trolox (100 µM) for 4 h, and autolysosomes formation was assessed by confocal microscopy. The data indicated that inhibition of ROS significantly reduced glucose starvation-induced autolysosomes formation and autophagic flux relative to vehicle-treated control cells (*P* < 0.001, **Figures 3C, D**). We confirmed the suppressive effect of Trolox on cellular ROS using DCFDA/H2DCFDA – Cellular Reactive Oxygen Species Detection Assay Kit and flow cytometry in SK-GT-4 cells (*P* < 0.05, **Figure 3E**) and FLO-1 cells (*P* < 0.01, **Figure 3F**) relative to their corresponding vehicle-treated control cells. Collectively, the results indicated that glucose starvation-induced activation of AMPK-ULK1 signaling and autophagic flux may be mediated by cellular ROS in EAC cells.

### AXL Positively Regulates Basal Cellular ROS Level in EAC Cells

**Figure 4:**
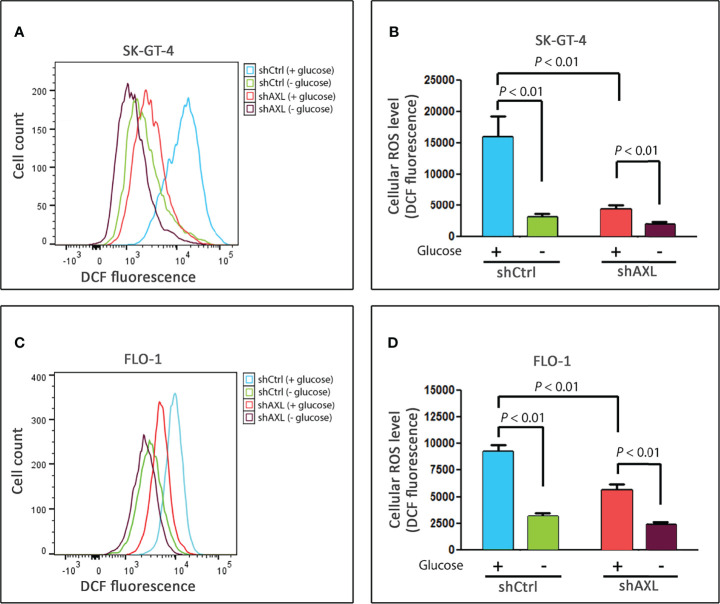
AXL is a positive regulator of basal cellular ROS levels in EAC cells. SK-GT-4-shCtrl and SK-GT-4-shAXL cells **(A**, **B)** or FLO-1-shCtrl and FLO-1-shAXL cells **(C**, **D)** were cultured in the presence or absence of glucose for 4 h, and then subjected to staining with DCFDA and followed by flow cytometry for detection of cellular ROS. Data are representative of three independent experiments and statistical significance was evaluated by one-way ANOVA followed by the Newman-Keuls *post hoc* test.

Based on our data that knocking down of AXL expression (**Figure 1**) or inhibition of cellular ROS (**Figure 3**) markedly suppressed glucose starvation-induced autophagic flux in EAC cells, we investigated if AXL mediates autophagy through regulation of ROS levels. SK-GT-4 and FLO-1 cells stably expressing control shRNA or AXL shRNA were cultured with or without glucose for 4 h and subjected to staining with CM‐H2DCFDA (detection of cellular ROS) or MitoSOX (detection of mitochondrial ROS) reagents and flow cytometry. The data showed that knockdown of AXL expression, under non-starving conditions, significantly decreased basal cellular ROS levels in SK-GT-4 cells (*P* < 0.01, **Figures 4A, B**) and FLO-1 cells (*P* < 0.01, **Figures 4C, D**), without a significant effect on basal mitochondrial ROS levels in SK-GT-4 cells (**Figures S2A, B**) and FLO-1 cells (**Figures S2C, D**) relative to their corresponding control cells. Notably, in response to glucose starvation, there was a significant decrease in cellular and mitochondrial ROS levels in SK-GT-4-shCtrl and SK-GT-4-shAXL cells (*P* < 0.01, **Figures 4A, B** and **Figures S2A, B**), and FLO-1-shCtrl and FLO-1-shAXL cells (*P* < 0.01, **Figures 4C, D** and **Figures S2C, D**) relative to their corresponding control cells. Together, the data indicate that AXL is a positive regulator of basal cellular ROS production in EAC cells. This suggests that cells exhibiting higher levels of AXL-dependent basal cellular ROS are more sensitive to glucose starvation-induced autophagy than those with lower levels.

### AXL Expression Is Required for Metformin-Induced Autophagy and Apoptosis in EAC Cells

**Figure 5:**
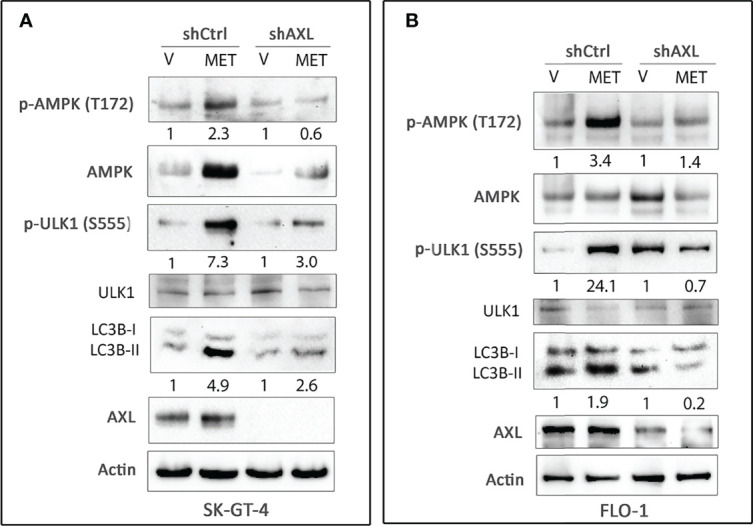
Knockdown of AXL expression abrogates metformin-induced autophagy in EAC cells. Western blot analysis of p-AMPK (T172), AMPK, p-ULK1 (S555), ULK1, LC3B, and AXL proteins in whole cell lysates from SK-GT-4-shCtrl and SK-GT-4-shAXL cells **(A)** and FLO-1-shCtrl and FLO-1-shAXL cells **(B)** following treatment with vehicle or metformin (15 mM or 10 mM) for 2 h. Gel loading was normalized for equal β-actin. Data are representative of three independent experiments.

**Figure 6:**
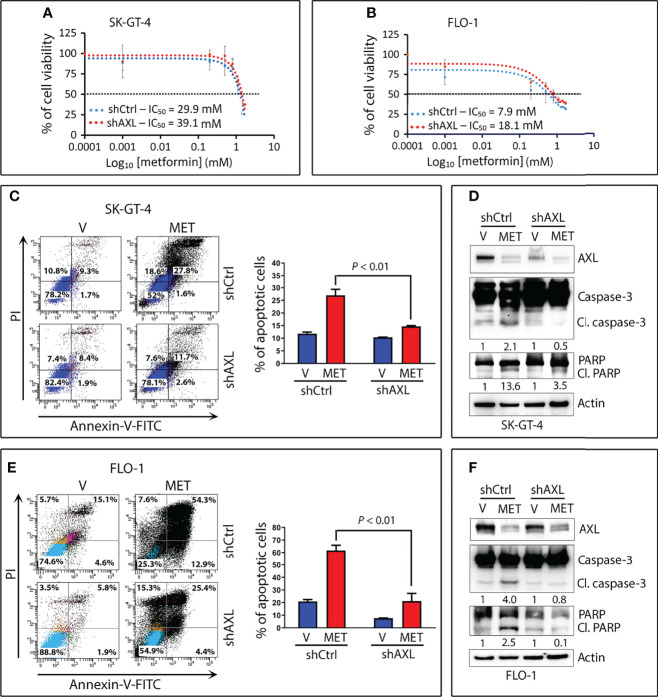
Downregulation of AXL expression attenuates metformin-induced apoptotic cell death in EAC cells. SK-GT-4-shCtrl and SK-GT-4-shAXL cells **(A)** or FLO-1-shCtrl and FLO-1-shAXL cells **(B)** were subjected to CCK-8 cell viability assay following treatment with the indicated metformin concentrations for 72 h. **(C)** SK-GT-4-shCtrl and SK-GT-4-shAXL cells were treated with vehicle or metformin (15 mM) for 96 h. Apoptosis was determined by Annexin-V/FITC staining and FACS analysis. **(D)** Immunoblot analysis of AXL, caspase-3, and PARP proteins in cell lysates from SK-GT-4-shCtrl and SK-GT-4-shAXL cells treated with vehicle or 15 mM metformin for 96 h. **(E)** Apoptosis in FLO-1-shCtrl and FLO-1-shAXL cells after treatment with vehicle or 10 mM metformin for 72 h was assessed as in panel **(C)**. **(F)** Western blot analysis of AXL, caspase-3, and PARP proteins in FLO-1-shCtrl and FLO-1-shAXL cells treated with vehicle or 10 mM metformin for 72 h. Gel loading was normalized for equal β-actin. Data are representative of three independent experiments and statistical analysis was evaluated by one-way ANOVA followed by the Newman-Keuls *post hoc* test.

Based on our data that AXL mediates glucose starvation-induced autophagy, we investigated the role of AXL in autophagy induced by metformin in EAC cells. We found that knockdown of AXL expression attenuates p-AMPK (T172), p-ULK1 (S555), and LC3B-II protein levels in SK-GT-4 cells (**Figure 5A**) and FLO-1 cells (**Figure 5B**) relative to control cells in response to treatment with metformin (15 mM or 10 mM, respectively) for 2 h. We next investigated whether the effect of metformin on cell survival depends on AXL expression. The cell viability assay data indicated that knockdown of AXL expression in SK-GT-4 cells (**Figure 6A**) and FLO-1 cells (**Figure 6B**) enhanced cell survival relative to control cells in response to a 72-hour treatment with metformin. In fact, AXL knockdown increased the metformin IC_50_ from 29.9 mM to 39.1 mM in SK-GT-4 cells (**Figure 6A**) and from 7.9 mM to 18.1 mM in FLO-1 cells (**Figure 6B**) relative to control cells. The data clearly indicated that knockdown of AXL expression decreases the sensitivity of cells to metformin, thereby increasing cell survival.

We next examined the induction of apoptosis by metformin and the implication of AXL expression in EAC cells. Using the Annexin-V-FITC and FACS analysis, we found that knockdown of AXL expression significantly decreased metformin-induced apoptosis events by 51.3% in SK-GT-4 cells (*P* < 0.01, **Figure 6C**) and by 55.6% in FLO-1 cells (*P* < 0.01, **Figure 6E**) relative to control cells. Consistently, metformin induced distinctly less cleaved caspase-3 and PARP protein levels in SK-GT-4-shAXL cells (**Figure 6D**) and FLO-1-shAXL cells (**Figure 6F**) than in vehicle-treated control cells. Collectively, the results demonstrated that AXL expression is required to mediate metformin-induced apoptosis in EAC cells. In a rescue experiment, we infected FLO-1-shAXL cells with control or AXL recombinant adenovirus followed by a 72-h treatment with metformin. We found that overexpression of AXL markedly sensitized cells to metformin (**Figure S3A**) and enhanced metformin-induced apoptosis relative to control cells, as indicated by increased cleaved caspase-3 and PARP protein levels (**Figure S3B**).

**Figure 7:**
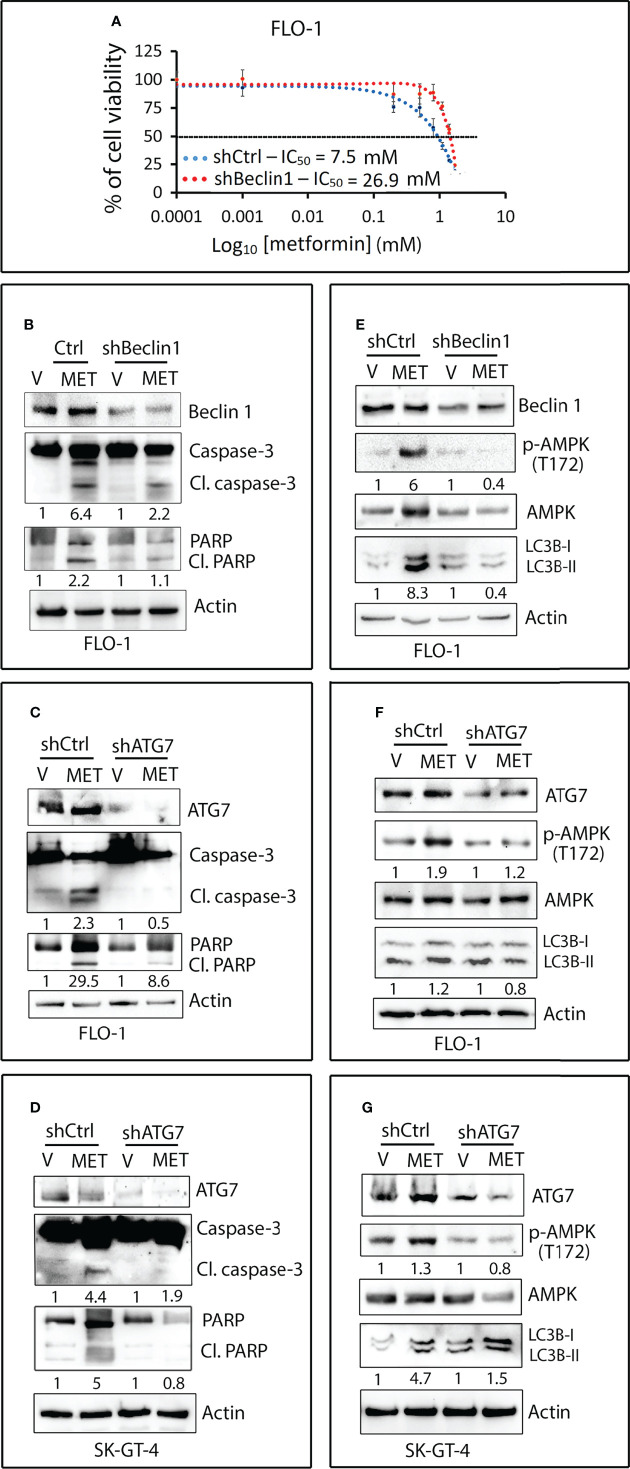
Metformin-induced apoptosis requires autophagy in EAC cells. **(A)** FLO-1 cells stably expressing control shRNA (shCtrl) or Beclin 1 shRNA (shBeclin1) were treated with increasing concentrations of metformin for 72 h and then subjected to CCK-8 cell viability assay. **(B)** Western blot analysis of Beclin 1, caspase-3, and PARP proteins in FLO-1-shCtrl and FLO-1-shBeclin1 cells treated with vehicle or 10 mM metformin for 72 h. **(C)** Immunoblotting of ATG7, Caspase-3, and PARP proteins in FLO-1-shCtrl and FLO-1-shATG7 cells treated with vehicle or 10 mM metformin for 72 h. **(D)** Western blot analysis of ATG7, caspase-3, and PARP proteins in SK-GT-4-shCtrl and SK-GT-4-shATG7 cells treated with 15 mM metformin for 96 h. **(E)** Western blot analysis of Beclin 1, p-AMPK (T172), AMPK, and LC3B proteins in FLO-1-shCtrl and FLO-1-shBeclin1 cells treated with vehicle or 10 mM metformin for 2 h. **(F)** Western blot analysis of ATG7, p-AMPK (T172), AMPK, and LC3B proteins in FLO-1-shCtrl and FLO-1-shATG7 cells treated with vehicle or 10 mM metformin for 2 h. **(G)** Immunoblotting of ATG7, p-AMPK (T172), AMPK, and LC3B proteins in SK-GT-4-shCtrl and SK-GT-4-shATG7 cells treated with 15 mM metformin for 2 h. Gel loading was normalized for equal β-actin.

We investigated if metformin-induced apoptosis depends on autophagy in EAC cells. The results showed that knockdown of the key mediator of autophagy Beclin1 in FLO-1 cells markedly increased cell survival relative to control cells in response to metformin (**Figure 7A**). Western blot analysis data indicated that knockdown of Beclin1 or ATG7 in FLO-1 cells (**Figures 7B, C**) and ATG7 in SK-GT-4 cells (**Figure 7D**) markedly reduced apoptosis in response to metformin, as indicated by a decrease in cleaved caspase-3 and PARP proteins (markers of apoptosis) relative to control cells. We confirmed that knockdown of Beclin1 or ATG7 in FLO-1 cells (**Figures 7E, F**) and ATG7 in SK-GT-4 cells (**Figure 7G**) suppresses metformin-induced autophagy, indicated by decreased p-AMPK (T172) and LC3B-II protein levels relative to corresponding control cells.

### AXL Expression Sensitizes Xenografted Tumors to Metformin *In Vivo*

**Figure 8:**
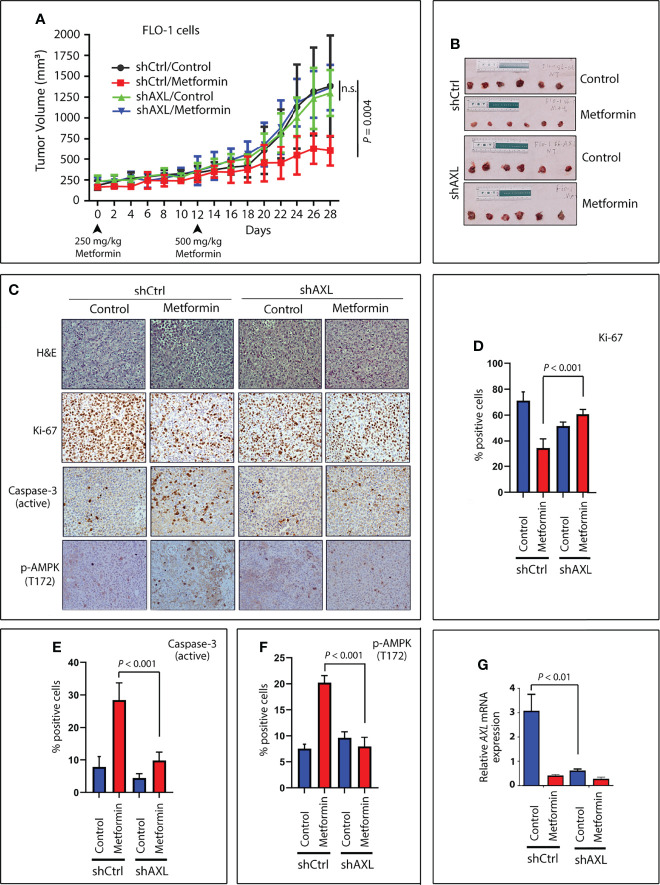
Expression of AXL sensitizes tumors to metformin in a xenograft mouse model. Four groups (10 mice per group) of Rag2 mice were injected on both hip flanks with FLO-1-shCtrl (two groups, no treatment and treatment) or FLO-1-shAXL cells (two groups, no treatment and treatment). When tumors reached a size of approximately 250 mm^3^, the mice were randomized and not treated or treated by oral gavage with metformin (250 mg/kg) daily for 12 days. The metformin dose was then increased to 500 mg/kg daily until the end of the experiment (day 28). **(A)** Treatment with metformin significantly reduced tumor size in shCtrl cells (*P* = 0.004) relative to nontreated tumors. Knockdown of AXL expression (shAXL cells) completely abrogated the suppressive effect of metformin on tumor growth. **(B)** Representative images of endpoint xenografts depicting the effect of metformin on tumor size. Images (200x magnification) of H&E staining **(C)** and IHC analysis for expression for Ki-67, a marker of proliferation **(C**, **D)**; cleaved caspase-3, a marker of apoptosis **(C**, **E)**; and p-AMPK (T172), a marker of autophagy **(C**, **F)** in representative endpoint tumors of all metformin-treated and non-treated animal groups. **(G)** Real-time PCR data depicting relative AXL mRNA expression in representative endpoint tumors of all the animal groups. n.s., not significant.

To investigate whether AXL expression provides a clinical advantage in the treatment of tumors with metformin, we used a tumor xenograft mouse model of FLO-1-shCtrl or FLO-1-shAXL cells. Xenografts were allowed to grow and reach 250 mm^3^ in size. After randomization, the mice were not treated or treated daily with metformin. Metformin at a dose of 500 mg/kg significantly reduced tumor growth of FLO-1-shCtrl cells relative to non-treated mice (*P* = 0.004, **Figures 8A, B**) without any adverse toxicity. Conversely, the metformin treatment had no significant effects on tumor growth in FLO-1-shAXL cells as compared to non-treated mice (**Figures 8A, B**), indicating that AXL expression is required for mediating the suppressive effect of metformin on tumor growth. Consistent with these results, IHC analysis of endpoint tumor xenografts indicated that metformin significantly induced lower Ki-67 (a marker of cell proliferation) and higher cleaved caspase-3 (a marker of apoptosis) protein levels in FLO-1-shCtrl than FL-1-shAXL tumors (*P* < 0.001, **Figures 8C–E**). Additionally, the data showed that metformin significantly induced higher p-AMPK (T172) protein expression in FLO-1-shCtrl than FLO-1-shAXL tumors (*P* < 0.001, **Figures 8C, F**). The results indicated that p-AMPK (T172) protein level positively correlates with increased apoptosis and low tumor growth rates in response to metformin *in vivo*. We confirmed the knockdown of AXL expression in FLO-1-shAXL tumors by real-time PCR (*P* < 0.01, **Figure 8G**). Collectively, the results demonstrated that AXL expression is required to sensitize tumors to the suppressive effects of metformin on growth and proliferation *in vivo*.

## Discussion

Previous studies implicated AXL in the regulation of autophagy in NSCLC cells (15) and mouse macrophages (14) but the underlying molecular mechanism remains poorly understood. In this study, we investigated the role of AXL in the regulation of autophagy in EAC cells and confirmed that AXL is a key mediator of glucose starvation-induced autophagy. We demonstrated that pharmacological inhibition or genetic knockdown of AXL significantly decreases autophagic flux. Our data indicated that AXL expression is required for the formation of autolysosomes and accumulation of LC3-II protein levels induced by glucose starvation in EAC cells. Mechanistic investigations revealed a novel finding that induction of autophagy by glucose starvation requires activation of AMPK that depends on AXL expression. In fact, genetic knockdown of AXL expression suppresses glucose starvation-induced phosphorylation of AMPK and ULK1 and accumulation of LC3B-II proteins. Under glucose deprivation conditions, AMPK induces autophagy by directly activating ULK1 by phosphorylation (42). ROS have been shown to directly activate AMPK through S-glutathionylation of cysteines on the AMPKα and β subunits (43) and induce autophagy through the AMPK signaling pathway (44). Our data confirmed that cellular ROS mediate activation of AMPK-ULK1 signaling pathway and autophagic flux by glucose starvation. Interestingly, we found that AXL positively regulates basal cellular but not mitochondrial ROS, suggesting that AXL mediates autophagy through regulation of cellular ROS. Although we have not elucidated the underlying mechanism, it is plausible that AXL expression increases basal cellular ROS levels through upregulation of NADPH oxidases (NOXs), which are one of the main sources of cellular ROS production (45). Additionally, abundance of peroxisomes and peroxisomal fatty acid oxidation-related pathway have been associated with increased hepatic cellular ROS levels in a non-alcoholic fatty liver disease (NAFLD) mouse model (46). Future studies will be required to determine the mechanism by which AXL regulates cellular ROS in EAC cells. Our results indicate that AXL-dependent high basal cellular ROS levels enhance autophagy through activation of AMPK-ULK1 signaling in cancer cells in response to metabolic stress.

Metformin, a common diabetes drug, inhibits mitochondrial complex I that suppresses oxidative phosphorylation leading to reduction of cellular energy levels (47) as well as activation of AMPK (48) and autophagy (49). Similar to the findings from the glucose starvation model of autophagy, we found that AXL expression is required for mediating metformin-induced activation of AMPK-ULK1 signaling and autophagy in EAC cells. Metformin reduces growth and proliferation of cancer cells through inhibition of cell division, which is a highly energy-consuming process (50, 51). Once activated by metformin, AMPK promotes compensatory catabolic processes such as glycolysis and fatty acid beta oxidation (52). We demonstrated that AXL expression sensitizes cancer cells to the suppressive effect of metformin on cell survival, leading to apoptosis. The molecular mechanism by which metformin induces apoptosis at a cellular level remains poorly understood. Therefore, we investigated if metformin-induced apoptosis depends on autophagy in EAC cells. We demonstrated that the disruption of autophagy by genetic knockdown of Beclin1 or ATG7 autophagy proteins attenuates metformin-induced autophagy and apoptosis. Interestingly, the knockdown of Beclin-1 or ATG7 blocked the metformin-induced phosphorylation of AMPK, the upstream inducer of autophagy, suggesting a possible crosstalk between AMPK and autophagy proteins. Further studies will be required to elucidate the underlying signaling mechanism. In contrast to EAC cells, pharmacological or genetic inhibition of autophagy sensitizes ESCC cells to metformin-induced apoptosis through downregulation of STAT3 signaling, implying the pro-survival function and nature of autophagy in ESCC cells (30). This report and our data confirm the cell type-dependent function of autophagy induced by metformin. Furthermore, while AXL expression enhances the pro-apoptotic effect of metformin in EAC cells, this metformin-induced effect is attenuated by AXL expression in ovarian cancer cells (31). Taken together, our findings strongly suggest that AXL promotes metformin-induced apoptosis through an autophagy-dependent mechanism in EAC cells. Our data indicate that metformin-induced autophagy exhibits a pro-apoptotic function in EAC cells but may play a pro-survival role in other types of cancer cells (30, 31).

Metformin normalizes blood glucose levels and exhibits anti-cancer properties in patients, making this drug a popular choice in clinical studies for repurposing it as a therapeutic agent against cancer (53). Moreover, metformin may prevent the development of cancer as indicated by retrospective clinical studies showing that diabetic patients receiving metformin have a decreased incidence of cancer (32). We investigated whether AXL expression promotes metformin-induced suppression of tumor growth *in vivo* using a xenograft mouse model of EAC. We demonstrated that AXL expression is highly predictive of the suppressive effect of metformin on tumor growth. Thus, AXL could be a valuable biomarker to identify the tumors that are sensitive to metformin in clinical studies. Development of AXL as a biomarker of drug response could be used to personalize metformin-based cancer therapy. Currently, there have been dozens of randomized clinical trials assessing metformin for cancer therapy. Therefore, targeting the sensitive tumors based on AXL expression may inform the selection of patients with EAC for future clinical trials to evaluate metformin.

## Materials and Methods

### Cell Lines and Reagents

The human esophageal adenocarcinoma cancer cell line, SK-GT-4, was purchased from Sigma-Aldrich (St. Louis, MO, USA). FLO-1 cells were provided by Dr. David Beer (University of Michigan, Ann Arbor, MI, USA). Both FLO-1 and SK-GT-4 cells were maintained in Dulbecco’s modified eagle’s medium (DMEM (11965-092), GIBCO, Carlsbad, CA, USA) supplemented with 5% fetal bovine serum (FBS) (A4766801, GIBCO) and 1% penicillin/streptomycin (15140-122, GIBCO) in a humidified atmosphere of 5% CO2 at 37^0^C. DMEM without glucose (11966-025) was obtained from GIBCO. All cell lines were authenticated with short tandem repeat (STR) profiling (Genetica DNA Laboratories, Burlington, NC, USA) and confirmed free of mycoplasma by MycoSEQ Mycoplasma Detection Kit (4460623, ThermoFisher Scientific, Waltham, MA, USA). Trolox (6-hydroxy-2,5,7,8-tetramethylchroman-2-carboxylic acid, 53188-07-1) and Chloroquine diphosphate salt (50-63-5) were obtained from Sigma-Aldrich. DCFDA/H2DCFDA - Cellular Reactive Oxygen Species Detection Assay (ab113851) and MitoSOX™ Red Mitochondrial Superoxide Indicator kits (ab219943) were purchased from ABCAM (Cambridge, United Kingdom). Metformin (NDC 50228-105-01) was obtained from SciGen Pharmaceuticals, Inc. (Hauppauge, NY, USA). The LC3B (D11) XP^®^ (#3868, 1:1000 dilution), AXL (C44G1) (#4566, 1:1000 dilution), p-AMPKα (T172) (#2531, 1:1000 dilution), AMPKα (#2532, 1:1000 dilution), p-ULK1 (S555) (D1H4) (#5869, 1:1000 dilution), ULK1 (D8H5) (#8054, 1:1000 dilution), Caspase-3 (#9662, 1:1000 dilution), PARP (46D11) (#9532, 1:1000 dilution), Atg7 (D12B11) (#8558, 1:1000 dilution), Beclin-1 (#3495, 1:1000 dilution), and β-Actin (13E5) (#4970, 1:1000 dilution) antibodies were purchased from Cell Signaling Technology (Danvers, MA, USA). The p-AXL (Y779) (#AF2228, 1:500 dilution) Antibody was purchased from R&D Systems (Minneapolis, MN, USA). Secondary HRP-linked goat anti-rabbit (W4011, 1:4000 dilution) and anti-mouse (W4021, 1:4000 dilution) antibodies were obtained from Promega Corporation (Madison, WI, USA).

### Knockdown of Gene Expression by Short Interfering RNA

For stable knockdown of AXL, BECN1 (Beclin 1), or ATG7 gene expression, non-target shRNA control (SHC016V-1EA) or a pool of five validated shRNA lentivirus particles (Sigma-Aldrich) for each gene (*AXL*: TRCN0000000572, TRCN0000000573, TRCN0000000576, TRCN0000195353, and TRCN0000194971; *BECN1*: TRCN0000033549, TRCN0000033550, TRCN0000033551, TRCN0000033552, and TRCN0000033553; *ATG7*: TRCN0000007584, TRCN0000007586, TRCN0000007587, TRCN0000007585, and TRCN0000007588) were used to transduce FLO-1 and SK-GT-4 EAC cells in the presence of Polybrene (4 µg/ml; Sigma-Aldrich). Puromycin (1 μg/ml, ant-pr-1, Invitrogen, Carlsbad, CA, USA) was added after 48 h from transduction for 2 weeks of selection.

### Monitoring Autophagic Flux With Confocal Microscopy in EAC Cells

Tandem fluorescent-tagged LC3 (mRFP-EGFP-LC3) is an established assay for evaluating autophagic flux based on different pH stability of mRFP and EGFP fluorescent proteins. Autophagosomes are indicated by yellow fluorescent puncta, resulting from merging green (EGFP) and red (mRFP) fluorescence. In autophagosomes, EGFP and mRFP proteins are stable in less acidic environment (pH higher than 5). However, in autolysosomes, generated by fusion of autophagosomes with acidic lysosomes, are indicated by red puncta. Under acidic environment (pH between 4 and 5) EGFP protein is degraded and green fluorescence is quenched, but mRFP protein and red fluorescence are stable. The number of red puncta per cell, indicating autolysosomes at which the final autophagic degradative step occurs, is a measure of autophagic flux. Cells were transfected with Tandem fluorescent-tagged LC3 (mRFP-EGFP-LC3) (ptfLC3) construct using Lipofectamine-2000. ptfLC3 (Addgene plasmid # 21074) was a gift from Tamotsu Yoshimori (54). After 48 h, cells were selected with 1.5 µg/ml puromycin (Invitrogen) for 2 weeks. For autophagic flux experiments, mRFP-EGFP-LC3-expressing cells were cultured for 4 h either in complete growth medium with glucose (4.5 g/L) or medium without glucose to induce autophagy. Confocal microscopy analysis was then used to measure formation of autolysosomes by monitoring the number of RFP fluorescent signals (red puncta) within cells.

### The Measurement of Cellular and Mitochondrial ROS Levels

Cells (4x10^5^ per well) were seeded into 6-well plates in complete medium. The next day, cells were cultured with either the complete medium or medium without glucose for 4 h. For measurement of cellular ROS levels, cells were incubated with 2.5 μM DCFDA for 30 min before analysis using a Becton Dickinson FACScan analytic flow cytometer. For measurement of mitochondrial ROS levels, the cells were incubated with 2.5 μM MitoSOX for 30 min before the flow cytometer analysis. Cells were gated using forward scatter and side scatter to remove debris and dead cells, and 10^4^ live cell events were recorded. To quantify changes in DCFDA or mitoSOX signal, mean fluorescent intensity after gating was used.

### Cell Viability Assay

Cell Counting Kit-8 (CCK-8) (CK08, Dojindo Laboratories, Kumamoto, Japan) was used to measure the cell viability in response to metformin treatment following the manufacturer’s protocols. Briefly, cells (5 × 10^3^ cells/well) were seeded in 96-well plates and cultured for 24 h. The cells were then treated with metformin with a range of concentrations from 0 – 100 mM for 72 h. After cells incubation with CCK-8 solution and determination of the absorbance at 450 nm, cell viability was shown as a percentage of that of the untreated cells. Six replicates were used for each condition. The IC50 curves were calculated using the GraphPad Prism 9.3.1.

### Apoptosis Assay

Cells (10^5^ cells per well) were seeded in 6-well culture plates. The following day, cells were treated with vehicle or metformin (10 – 15 mM) for 4 – 5 days. Apoptosis was evaluated as described previously (8). Briefly, cells were harvested and stained with Annexin-V fluorescein isothiocyanate (FITC) using TACS Annexin V-FITC Apoptosis Detection Kit (4830-250-K, R&D Systems) and propidium iodide (PI) (P4170, Sigma-Aldrich). The samples were then subjected to fluorescence-activated cell sorting (FACS) analysis by a flow cytometer (Becton Dickinson, Franklin Lakes, NJ, USA). Apoptotic cell death was determined by counting cells that stained positive for Annexin-V FITC and negative for PI (early apoptosis) in addition to cells that are positive for both Annexin-V FITC and PI (late apoptosis).

### Quantitative RT-PCR

Total RNA from frozen xenograft tissue sections was extracted using TRIzol reagent (15596018, Invitrogen) and cDNA was synthesized using a Sensi-FAST cDNA Synthesis Kit (BIO-65053, Bioline, Taunton, MA, USA), and the quantitative reverse transcriptase PCR (qRT-PCR) was performed as described previously (8). Briefly, the qRT-PCR was performed with a Bio-Rad CFX Connect Real-time System in a 10-µL reaction volume using iQ SYBR Green Supermix (1708880, Bio-Rad Laboratories Inc., Hercules, CA, USA) with the gene-specific primers. The threshold cycle number was determined by CFX MANAGER™ software version 3.0 (Bio-Rad). Reactions were performed in triplicate, and the threshold cycle numbers were averaged. The data were normalized to the *HPRT1* housekeeping gene. The relative mRNA expression levels were calculated according to the formula 2(RT – ET)/2(Rn – En).

### Western Blot Analysis

Preparation of whole-cell cell lysates and Western blot analysis were performed as described previously (9). Briefly, cells were scraped and centrifuged at 4^0^C. Pellets were lysed in RIPA lysis buffer with 1 x Halt protease and phosphatase inhibitors cocktail (sc-364162, Santa Cruz Biotechnology). Protein concentrations were measured using Bio-Rad protein Assay (Bio-Rad). Proteins were separated by sodium dodecyl sulfate polyacrylamide gel electrophoresis (SDS/PAGE) and transferred to nitrocellulose membranes (0.45 µm, 1620115, Bio-Rad). Membranes were probed with specific primary antibodies, followed by horseradish peroxidase (HRP)-conjugated secondary antibodies (Promega). Protein bands were visualized using Immobilon Western Chemiluminescent HRP Substrate detection reagent (P90720, Sigma-Aldrich). Images were taken on the ChemiDoc XRS System (Bio-Rad) and the protein bands intensities were quantified by NIH ImageJ software. The relative values for various proteins regulated by autophagy induced by glucose starvation or metformin were calculated and shown as a ratio relative to control treatments.

### Fluorescence Microscopy

Cells stably expressing mRFP-GFP tandem fluorescent-tagged LC3 plasmid reporter were seeded on glass bottom culture dishes (MatTek Corporation) coated with fibronectin and grown to 80% confluence for 24 h. Following glucose starvation alone or in combination with drug treatments, images of live cells were acquired using a laser scanning confocal microscope LSM 880 (Zeiss) with an oil immersion 60× NA 1.49 objective using fluorophore-specific lasers. Quantification of total number of autolysosomes (red puncta) from 30 to 50 cells from images of random microscopic fields was carried out manually using the Pen tool (Adobe Photoshop 2021).

### Immunohistochemistry

The endpoint xenograft tumors were isolated, fixed in formalin, and paraffin embedded. Tissue sections were processed for immunostaining and incubated overnight with p-AMPKα (T172) (40H9) (#2535, 1:200 dilution), Ki-67 (D2H10) (#9027, 1:200 dilution), or cleaved caspase-3 (Asp175) (5A1E) (#9664, 1:400 dilution) antibodies (Cell Signaling Technology). Next, the sections were counterstained with hematoxylin and subjected to IHC analysis as described previously (8).

### Tumor Xenograft Mouse Model

Four-week-old B6;129-Rag2tm1FwaII2rgtm1Rsky/DwlHsd (R2G2) female mice (Envigo RMS Division, Indianapolis, IN, USA) were randomized into four groups (10 mice per group). FLO-1-shCtrl (two groups, no treatment and drug treatment groups) or FLO-1-shAXL cells (two groups, no treatment and treatment groups). Cells (5 x 10^6^ cells per site), suspended in 200 µl DMEM/growth factor reduced Matrigel (354234, BD Biosciences) mixture (50% DMEM supplemented with 10% FBS and 50% Matrigel), were injected subcutaneously into the flank regions on both sides of each mouse. When tumors reached approximately 250 mm^3^ in size, the mice were either not treated (controls) or treated daily with metformin (250 mg/kg, formulated in PBS) by oral gavage for 12 days. The dose was then increased to 500 mg/kg daily until the end of the experiment (day 28). The tumor xenograft volume was determined as described previously (8). The animal protocol was approved by the Vanderbilt Institutional Animal Care and Use Committee.

### Statistical Analysis

The results were expressed as the mean with ± standard deviation (SD). The statistical significance of the studies was determined by one-way ANOVA followed by the Newman-Keuls *post hoc* test. All statistical analyses were performed using GraphPad Prism 8.0. Differences were considered to be statistically significant at *P* < 0.05.

## Data Availability Statement

The raw data supporting the conclusions of this article will be made available by the authors, without undue reservation.

## Ethics Statement

The animal study was reviewed and approved by Institutional Animal Care and Use Committee (IACUC) - Vanderbilt University Medical Center.

## Author Contributions

JH: design of experiments, generation and analysis of data, and writing the manuscript; SM: data analysis and writing the manuscript; NP: editing the manuscript and data analysis; ABa: generating data; SS: editing the manuscript; MW: editing the manuscript and analysis of data; ABe: funding, experimental design, data analysis, and writing the manuscript. All authors contributed to the article and approved the submitted version.

## Funding

The National Cancer Institute (NCI) of the National Institutes of Health (NIH) - RO1CA193219. Vanderbilt-Ingram Cancer Center (VICC) - P50CA95103.

## Conflict of Interest

The authors declare that the research was conducted in the absence of any commercial or financial relationships that could be construed as a potential conflict of interest.

## Publisher’s Note

All claims expressed in this article are solely those of the authors and do not necessarily represent those of their affiliated organizations, or those of the publisher, the editors and the reviewers. Any product that may be evaluated in this article, or claim that may be made by its manufacturer, is not guaranteed or endorsed by the publisher.

## Abbreviations

AMPK, AMP-activated protein kinase; ATG7, autophagy related 7; BE, Barrett’s esophagus; CQ, chloroquine; EAC, esophageal adenocarcinoma; ESCC, esophageal squamous cell carcinoma; LC3, microtubule-associated protein 1A/1B-light chain 3; ROS, reactive oxygen species; tfLC3, mRFP-GFP tandem fluorescent-tagged LC3 plasmid reporter; ULK1, UNC51-like Kinase 1.
