# Supplementary material for: AXL Promotes Metformin-Induced Apoptosis Through Mediation of Autophagy by Activating ROS-AMPK-ULK1 Signaling in Human Esophageal Adenocarcinoma
Source: Front Oncol. 2022 Jul 22;12:903874. doi: 10.3389/fonc.2022.903874 (PMC9354051; doi:10.3389/fonc.2022.903874)
Supplement: Supplementary file 1 [file DataSheet_1.pdf]

Supplemental Figures

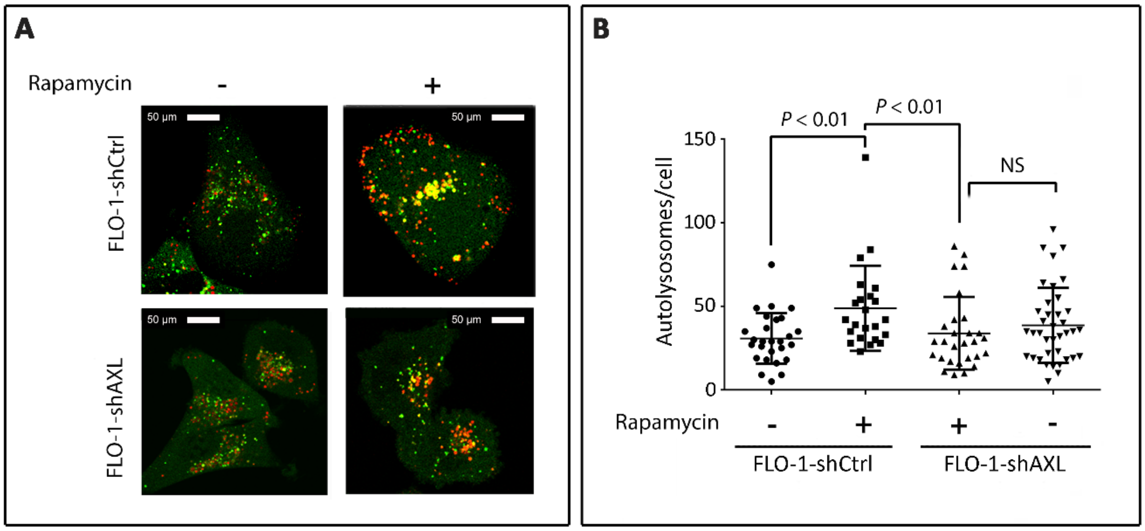

Figure S1. Hong et al

Supplemental Figure S1. Knockdown of AXL expression impairs rapamycin-induced autophagic flux in EAC cells. (**A**) FLO-1-shCtrl and FLO-1-shAXL cells stably expressing mRFP-GFP tandem fluorescent-tagged LC3 reporter (tfLC3) were cultured in the presence of 100 nM rapamycin (inhibitor of mTOR kinase) or vehicle for 4 h, and autolysosomes formation was assessed by confocal fluorescence microscopy. Representative confocal images (60x) of live cells depicting autophagosomes (green puncta) and autolysosomes (red puncta). (**B**) Quantification of total number of autolysosomes per cell in FLO-1-shCtrl versus FLO-1-shAXL. Data, which are shown as median ± SD, are representative of three independent experiments and statistical significance was evaluated by one-way ANOVA followed by the Newman-Keuls *post hoc* test . NS, statistically not significant.

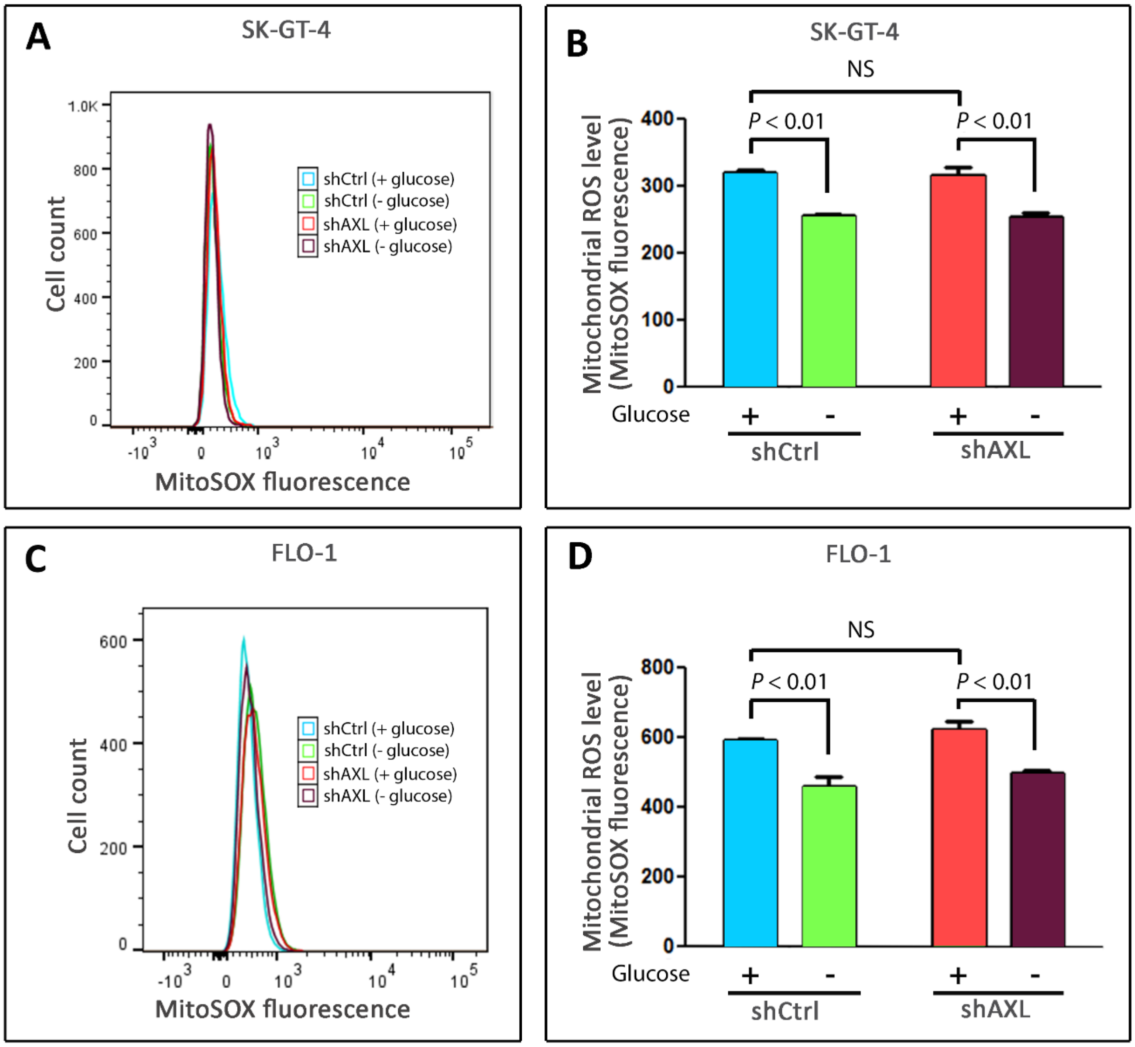

Figure S2. Hong et al

Supplemental Figure S2. Basal mitochondrial ROS level is not significantly affected by knockdown of AXL expression in EAC cells. SK-GT-4-shCtrl and SK-GT-4-shAXL cells (**A-B**) or FLO-1-shCtrl and FLO-1-shAXL cells (**C-D**) were cultured in the presence or absence of glucose for 4 h, and then incubated with 2.5 μM MitoSOX (Invitrogen) for 30 min and followed by flow cytometry analysis for measurement of mitochondrial ROS. Data are representative of three

independent experiments and statistical significance was evaluated by one-way ANOVA followed by the Newman-Keuls *post hoc* test. NS, statistically not significant.

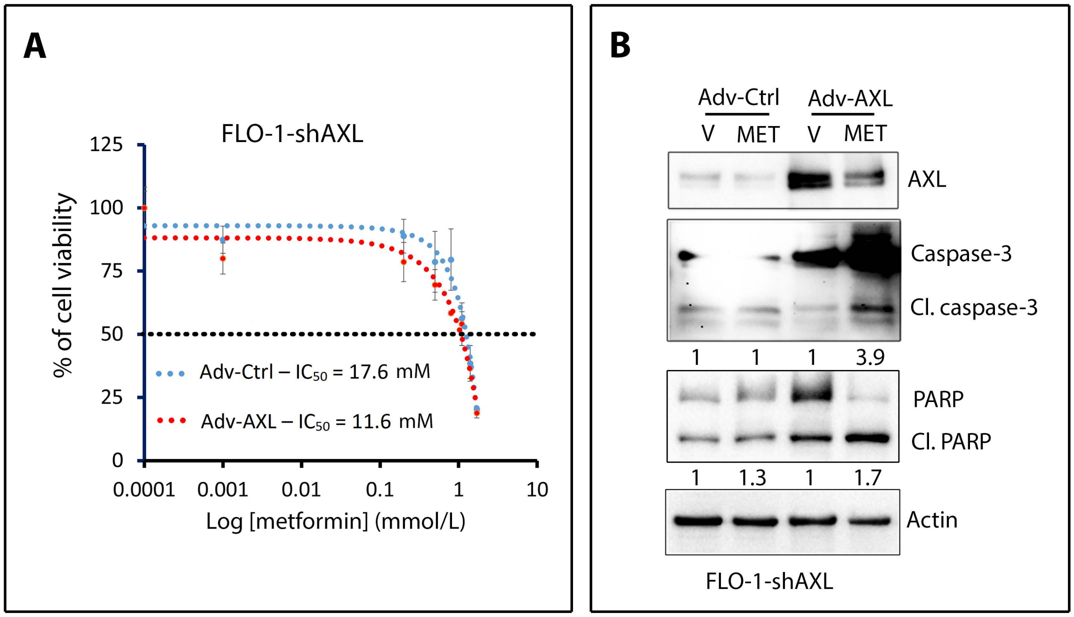

Figure S3. Hong et al

Supplemental Figure S3. Overexpression of AXL enhances sensitivity to metformin in EAC cells. (**A**) FLO-1-shAXL cells were infected with control or AXL recombinant adenoviruses (100 MOI), followed by a 72-hour treatment with increasing concentrations of metformin, and subjected to CCK-8 cell viability assay. (**B**) Western blot analysis of AXL, caspase-3, and PARP proteins in FLO-1-Adv-Ctrl and FLO-1-Adv-AXL cells treated with vehicle or 10 mM metformin for 72 h. Gel loading was normalized for equal β-actin. Data are representative of three independent experiments.

Supplemental methods

Generation of recombinant adenoviruses

The recombinant control and AXL-expressing adenoviruses were generated as described previously [1]. Briefly, the coding sequence of AXL from pcDNA3.1/AXL plasmid was subcloned into the adenoviral shuttle vector (pACCMV). The recombinant AXL-expressing adenovirus was generated by co-transfecting HEK-293 cells with the shuttle and backbone (pJM17) adenoviral plasmids using the Calcium Phosphate Transfection Kit (Applied Biological Materials Inc, Richmond, BC).

Supplemental references

1. Hong J, Peng D, Chen Z, Sehdev V, Belkhiri A. ABL regulation by AXL promotes cisplatin resistance in esophageal cancer. Cancer Res 2013; 73:331-40.
